# Association of Tourette Syndrome and Chronic Tic Disorder With Violent Assault and Criminal Convictions

**DOI:** 10.1001/jamaneurol.2022.0167

**Published:** 2022-03-21

**Authors:** David Mataix-Cols, Suvi Virtanen, Anna Sidorchuk, Lorena Fernández de la Cruz, Henrik Larsson, Paul Lichtenstein, Antti Latvala

**Affiliations:** 1 Centre for Psychiatry Research, Department of Clinical Neuroscience, Karolinska Institutet, Stockholm, Sweden; 2 Stockholm Health Care Services, Region Stockholm, Stockholm, Sweden; 3 Institute of Criminology and Legal Policy, University of Helsinki, Helsinki, Finland; 4 Department of Medical Epidemiology and Biostatistics, Karolinska Institutet, Stockholm, Sweden; 5 School of Medical Sciences, Örebro University, Örebro, Sweden

## Abstract

**Importance:**

Tic disorders are associated with multiple social adversities, but little is known about the experience of violent assault (including sexual assault) and criminality in this group.

**Objective:**

To establish if Tourette syndrome (TS) and chronic tic disorder (CTD) are associated with an increased risk of experiencing violent assault and criminal convictions.

**Design, Setting, and Participants:**

In this cohort study, all individuals living in Sweden at any time between January 1, 1973, and December 31, 2013, were identified from Swedish nationwide health and administrative registers. Cox proportional hazards regression models were used to estimate the risk of violent assault and criminal convictions among people with TS or CTD, compared with the general population and unaffected full siblings. Data analyses were conducted between September 1 and October 22, 2021.

**Exposures:**

The Swedish version of the *International Classification of Diseases, Eighth Revision *(*ICD-8*),* ICD-9*, and* ICD-10* diagnoses of TS or CTD in the Swedish National Patient Register.

**Main Outcomes and Measures:**

Records of sexual and nonsexual violent assault and death due to violent assaults were obtained from the National Patient Register and the Cause of Death Register, respectively. Convictions for violent and nonviolent criminal offenses were obtained from the Crime Register. Covariates included sex and birth year.

**Results:**

The study cohort included 13 819 284 individuals living in Sweden between 1973 and 2013. A total of 7791 individuals with TS or CTD were identified (median [IQR] age at first diagnosis, 13.4 [10.0-21.8] years; 5944 [76%] male). Compared with unaffected individuals from the general population, people with TS or CTD had a 2-fold increased risk of experiencing any violent assault (sexual and nonsexual) (adjusted hazard ratio [aHR], 2.21; 95% CI, 2.00-2.43), a 3-fold increased risk of violent convictions (aHR, 3.13; 95% CI, 2.92-3.36), and a 1.6-fold increased risk of nonviolent crime convictions (aHR, 1.62; 95% CI, 1.54-1.71). Among people with TS or CTD, 37.0% (114 of 308; 95% CI, 31.6%-42.4%) of individuals who had experienced violent assault also had a violent crime conviction, compared with 17.9% (16 067 of 89 920; 95% CI, 17.6%-18.1%) in the general population. Exclusion of individuals with attention-deficit/hyperactivity disorder and substance use disorders partially attenuated the associations. Similarly, within-sibling models attenuated but did not eliminate the associations (any violent assault: aHR, 1.32; 95% CI, 1.08-1.61; violent crime: aHR, 2.23; 95% CI, 1.86-2.67; nonviolent crime: aHR, 1.34; 95% CI, 1.20-1.50).

**Conclusions and Relevance:**

Results of this cohort study suggest that most individuals with TS or CTD are not assaulted nor are perpetrators of crime. However, individuals with TS or CTD diagnosed in specialist settings were more likely to both experience violent assault and be perpetrators of violence compared with the general population. The risk was highest in individuals with comorbid attention-deficit/hyperactivity disorder and substance use disorders. The increased risk found in specialty clinics will need to be better understood in the general population.

## Key Points

**Question:**

Are Tourette syndrome (TS) and chronic tic disorder (CTD) associated with an increased risk of experiencing violent assault and criminal convictions?

**Findings:**

In this cohort study including 7791 individuals with TS or CTD diagnosed in specialist settings and 13 811 493 unaffected individuals, individuals with TS or CTD had a 2.2-fold increased risk of experiencing violent assault (including sexual assault), a 3.1-fold increased risk of violent crime convictions, and a 1.6-fold increased risk of nonviolent crime convictions.

**Meaning:**

Findings suggest that most individuals with TS or CTD are not assaulted nor are perpetrators of crime; however, individuals with TS or CTD diagnosed in specialist settings were more likely to both experience violent assault and be perpetrators of violence.

## Teaser

This cohort study investigates whether Tourette syndrome or chronic tic disorder are associated with an increased risk of experiencing violent assault and criminal convictions.

## Introduction

Tourette syndrome (TS) and chronic tic disorder (CTD) often co-occur with other neuropsychiatric conditions, such as attention-deficit/hyperactivity disorder (ADHD),^1^ and are associated with multiple social adversities, including school failure, stigma, social rejection, difficulty establishing relationships, and suicidality.^2,3,4,5,6,7,8^ Most of what is known on the topic of bullying in TS or CTD originates from self- or parent-reported data.^3,6,9^ For example, in the 2016 to 2017 National Survey of Children’s Health, 56.1% of US children (104 of 186) with a parent-reported diagnosis of TS experienced bullying, 20.7% (39 of 186) experienced bullying perpetration, and 15.9% (30 of 186) experienced both.^6^ After adjusting for age, sex, and co-occurring psychiatric disorders, only the association of bullying remained statistically higher in children with TS compared with unaffected children.^6^

Much less is known about the experience of assault, such as sexual or other violent assaults causing serious injuries, and criminal offenses (ie, the perpetration of crime) in TS and CTD. Some individuals with highly comorbid TS or CTD experience sudden explosive outbursts,^9^ which may put them at higher risk to be assaulted or get criminal convictions. Case studies have described individuals with TS or CTD who had trouble with the law, sometimes owing to misunderstanding of the disorder’s symptoms, such as socially inappropriate behavior, obscene gestures, or rage attacks.^10,11,12,13^ A study of 217 tic disorder cases diagnosed in child and adolescent mental health services in Stockholm, Sweden, found that young people with TS had 2.2-fold higher odds of violent criminality than matched controls.^14^ Another Swedish study reported a greater than 2-fold higher risk of substance use–related criminal convictions in 7832 individuals with TS or CTD, compared with unaffected individuals from the general population.^15^ Taken together, the limited available evidence suggests that at least some individuals with TS or CTD may be more likely to both experience violent assault and be perpetrators of crime. Because most people with TS or CTD have psychiatric comorbidities^1,16^ and psychiatric disorders are robustly associated with both assault experience and perpetration of crime,^17^ it is important to understand if the associations are independent of psychiatric comorbidities.

It is widely believed that much of the social adversity experienced by individuals with TS or CTD is at least partially caused by misconceptions about the disorder by parents, teachers, peers, health care professionals, and the wider community.^5,18^ However, twin and family studies have reported that psychiatric disorders, violent crime, and the experience of violence aggregate in families^19,20,21^ and may share genetic risk factors.^22,23^ Therefore, it is critical that familial confounding is taken into account when studying the association between neuropsychiatric disorders, assault experience, and crime.^24^

We analyzed data from a large specialty cohort of prospectively observed patients with TS or CTD to (1) estimate the risk of different types of violent assault and criminal convictions in individuals with TS or CTD at the population level, (2) establish whether the association between TS or CTD, violent assault experience, and crime is explained by psychiatric comorbidity, and (3) investigate whether the association is explained by familial factors shared by siblings.

## Methods

### Data Sources

This cohort study used the unique Swedish national identification number^25^ to link Swedish nationwide health and administrative registers. Data on demographic characteristics, migration, and deaths were extracted from the Total Population Register^26^ and the Cause of Death Register.^27^ Information on diagnoses given in both inpatient (from 1969, with nationwide coverage for psychiatric disorders from 1973) and outpatient specialist services (since 2001) were retrieved from the National Patient Register. In this register,^28^ diagnoses are coded using the Swedish version of the *International Classification of Diseases, Eighth Revision *(*ICD-8*),* ICD-9*, and *ICD-10*. Medication data were collected from the Prescribed Drug Register, which covers all prescribed medications dispensed in pharmacies in Sweden since July 2005.^29^ Convictions for criminal offenses were collected from the nationwide Crime Register, with coverage since 1973. The Multigeneration Register allows for linking individuals born in Sweden from 1932 or registered as living in the country since 1961, with their parents, and we used the linkage to identify full siblings within the cohort.^30^

This study was approved by the Regional Ethical Review Board in Stockholm, and the need for informed consent was waived owing to the use of deidentified patient data. This study followed the Strengthening the Reporting of Observational Studies in Epidemiology (STROBE) reporting guidelines.

### Study Population and Exposure Variables

The study cohort consisted of all individuals living in Sweden at any time between January 1, 1973, and December 31, 2013. We collected lifetime diagnoses of TS or CTD based on the Swedish versions of the *ICD-8 *(code 306.2),* ICD-9 *(code 307C), and* ICD-10 *(transient tic disorder, code F95.0; chronic motor or vocal tic disorder, code F95.1; Tourette syndrome, code F95.2; other tic disorders, code F95.8; or unspecified tic disorder, code F95.9). In line with prior research,^2,31,32^ exposure status was ascertained by using an algorithm that minimizes the inclusion of individuals with only transient tics using a minimum age of 3 years for diagnosis to avoid diagnostic misclassification. The Swedish *ICD* codes for TS or CTD diagnoses have excellent validity and reliability.^33^

The cohort was followed up from January 1, 1973, immigration to Sweden, or birth (age 15 years for conviction outcomes), whichever occurred last, until the date of the outcome, emigration from Sweden, death, or end of the study period (ie, December 31, 2013), whichever occurred first. People with follow-up ending before age 3 years were excluded. Data on race and ethnicity are not collected in the Swedish registers, and these data were not required by the funders of this study.

### Outcomes

We defined violent assault as *ICD* diagnoses of injuries owing to assaults in the National Patient Register, as well as death resulting from an assault in the Cause of Death Register. Further, we examined sexual assault separately, which was defined as *ICD* diagnoses of sexual assault and abuse in the National Patient Register.

Criminal conviction outcomes from the Crime Register included violent crimes (eg, assault, robbery, sexual crimes, illegal threats) and nonviolent crimes, further divided into 4 subgroups: (1) alcohol- and drug-related crimes, including driving under the influence of alcohol or drugs and violations of the Narcotic Drugs Act, such as possession, manufacturing, trafficking, or sales of narcotics; (2) traffic-related crimes (eg, reckless driving, hit-and-run offenses, causing death or injury by driving, and moving violations); (3) property crimes (eg, theft, burglary); and (4) other nonviolent crimes. The minimum age for criminal responsibility in Sweden is 15 years. *ICD* codes are shown in eTable 1 in the Supplement.

### Psychiatric Comorbidities

To investigate whether psychiatric comorbidities contributed to the associations under study, we included lifetime diagnoses of the following groups of psychiatric disorders from the National Patient Register: (1) ADHD, (2) anxiety- and stress-related disorders, (3) autism spectrum disorders, (4) conduct disorders, (5) depressive disorders, (6) obsessive-compulsive disorder, (7) psychotic disorders (schizophrenia spectrum disorders and bipolar disorder), and (8) substance use disorders. Individuals were also classified as having ADHD if they had ever been dispensed stimulant medication, which is used almost exclusively for the treatment of ADHD. *ICD* and medication codes are listed in eTable 2 in the Supplement. Many of these diagnostic codes have been validated with satisfactory results.^33,34,35,36,37,38^

### Statistical Analyses

#### Primary Analyses

For aim 1, we used Cox proportional hazards regression, with age in years as the underlying time scale, to estimate the association of TS or CTD with assault and criminal conviction outcomes. We compared the risk of any experienced assault and, separately, sexual and nonsexual violent assault, as well as violent and nonviolent criminal convictions, in individuals with TS or CTD with that of unaffected individuals in the general population. We present estimates both for the full sample and separately for men and women. For aim 2, we repeated the main analyses excluding different groups of comorbid psychiatric disorders one at a time to establish whether the association of TS or CTD with the experience of violent assault and convictions for criminal offenses was explained by psychiatric comorbidities.

For aim 3, we investigated the association of TS or CTD with the experience of assault and criminal convictions in a subsample of full siblings within the cohort, identified as individuals who share both biological parents. We used Cox proportional hazards regression models stratified by sibling clusters, which rule out all factors constant within full siblings (ie, on average 50% of genetic factors and all shared environmental influences, eg, parental socioeconomic status). Such stratified models use information from clusters with discordance in the exposure to estimate the associations of TS or CTD with the assault and criminality outcomes within families. We excluded monozygotic twins and twins with unknown zygosity from the analyses.

#### Additional Analyses

To ensure that the observed associations were not biased owing to lack of data coverage (eg, exposure/outcome occurring before the start of the follow-up), we repeated the analyses for aim 1 in a subcohort of individuals born in Sweden after 1972 (ie, those who had complete follow-up from birth). Within this cohort, we also estimated the cumulative incidence of any assault experience and crime convictions for individuals with and without TS or CTD using Kaplan-Meier survival estimates under the assumption of no competing risks (estimated as 1 minus the Kaplan-Meier estimate of survival function). To investigate the potential overlap in assault experience and violent offenses, we calculated the proportion of those who experienced violent assault (both sexual and nonsexual) with those who also had a violent crime conviction, and vice versa, among people with TS or CTD and in the unaffected general population.

Because the National Patient Register does not contain information on TS or CTD severity, we repeated the analyses for aim 1 in a subcohort of individuals who had been diagnosed with TS or CTD at least twice after the age of 18 years, used as a proxy for severity and long-term persistence of TS or CTD into adulthood, and compared them with individuals without a TS or CTD diagnosis. This analysis excluded individuals diagnosed with TS or CTD who were no longer seen by a specialist after age 18 years as well as those who were evaluated only once after age 18 years. All models were adjusted for sex and birth year. Data management and analyses were performed between September 1 and October 22, 2021, using SAS, version 9.4 (SAS Institute) and Stata, version 15.1 (StataCorp), respectively.

## Results

### Descriptive Statistics

This cohort study included 13 819 284 individuals living in Sweden between 1973 and 2013. A total of 7791 individuals with TS or CTD were identified, of whom 5944 (76%) were male, and 1847 (24%) were female. The median (IQR) age at first TS or CTD diagnosis was 13.4 (10.0-21.8) years in the total cohort and 12.2 (9.5-16.7) years in those with follow-up from birth. Most people with TS or CTD had at least 1 comorbid psychiatric disorder (eTable 2 in the Supplement). The median (IQR) length of follow-up was 29.9 (16.8-41.0) years for violent assault outcomes and 18.4 (6.4-35.1) years for crime conviction outcomes.

### Risk of Violent Assault

**Figure 1.**
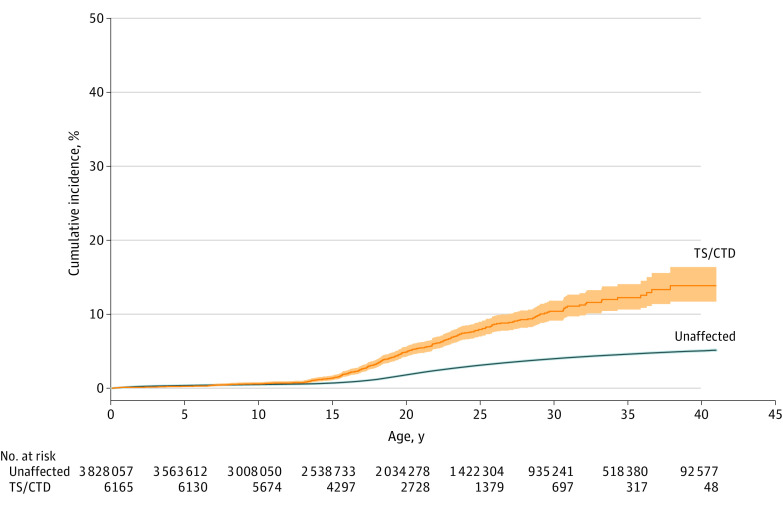
Any Violent Assault in Individuals With Tourette Syndrome (TS) or Chronic Tic Disorder (CTD) Cumulative incidence under the assumption of no competing risks estimated as 1 minus the Kaplan-Meier estimate of survival function for any violent assault in individuals with TS or CTD and unaffected individuals from the general population in the subsample of 3 834 222 individuals followed up from birth. The shaded areas indicate 95% CIs.

People with TS or CTD had a 2-fold increased risk of any violent assault, compared with unaffected individuals from the general population (sex- and birth year–adjusted hazard ratio [aHR], 2.21; 95% CI, 2.00-2.43). Similar estimates were obtained for both sexual and nonsexual assault (Table 1). Based on nonoverlapping CIs, women with TS or CTD had a higher relative risk of experiencing any assault than men. In terms of absolute risks, the cumulative incidence of any violent assault reached 14% by the end of follow-up at age 41 years among people with TS or CTD, compared with 5% in the unaffected general population (Figure 1).

**Table 1.** Association of Tourette Syndrome or Chronic Tic Disorder With Violent Assault and Criminal Convictions

Outcome	People with Tourette syndrome or chronic tic disorder, No. (%)^a^	Rate per 10 000 person-years^b^	Unaffected general population, No. (%)^c^	Rate per 10 000 person-years^b^	Sex- and birth year–adjusted HR (95% CI)
**Violent assault **					
Any	401 (5.2)	28.3	254 805 (1.8)	8.0	2.21 (2.00-2.43)
Men	309 (5.2)	28.3	146 000 (2.1)	9.2	1.90 (1.70-2.12)
Women	92 (5.0)	28.4	108 805 (1.6)	6.7	2.98 (2.43-3.66)
Sexual	59 (0.8)	4.0	45 594 (0.3)	1.4	2.79 (2.16-3.60)
Men	16 (0.3)	1.4	16 206 (0.2)	1.0	3.10 (1.90-5.07)
Women	43 (2.3)	11.5	29 388 (0.4)	1.7	4.40 (3.26-5.94)
Nonsexual^d^	354 (4.5)	24.3	214 187 (1.6)	6.6	2.14 (1.93-2.38)
Men	296 (5.0)	26.9	131 179 (1.9)	8.2	1.88 (1.68-2.11)
Women	58 (3.1)	16.9	83 008 (1.2)	5.0	2.57 (1.99-3.32)
**Conviction for criminal offense**					
Violent^e^	769 (13.1)	234.5	322 720 (2.6)	21.6	3.13 (2.92-3.36)
Men	691 (15.7)	310.5	284 990 (5.0)	40.2	3.04 (2.82-3.28)
Women	78 (5.3)	64.0	37 730 (0.6)	3.6	5.77 (4.62-7.21)
Nonviolent					
Any	1532 (26.1)	925.4	1 757 905 (14.3)	178.5	1.62 (1.54-1.71)
Men	1305 (29.7)	1178.1	1 341 662 (21.6)	308.2	1.61 (1.53-1.71)
Women	227 (15.4)	358.9	416 243 (6.8)	53.3	2.08 (1.82-2.37)
Alcohol- or drug-related	595 (10.1)	242.1	436 651 (3.5)	35.2	2.10 (1.94-2.27)
Men	532 (12.1)	310.9	381 178 (6.1)	64.0	2.03 (1.86-2.21)
Women	63 (4.3)	88.1	55 473 (0.9)	7.5	3.81 (2.97-4.88)
Traffic crime	557 (9.5)	176.4	769 370 (6.2)	50.2	1.45 (1.34-1.58)
Men	515 (11.7)	239.2	654 386 (10.6)	91.2	1.39 (1.28-1.52)
Women	42 (2.9)	35.5	114 984 (1.9)	10.6	1.88 (1.39-2.54)
Property crime	753 (12.8)	301.8	567 003 (4.6)	47.8	2.06 (1.92-2.21)
Men	613 (13.9)	364.9	381 366 (6.2)	74.8	2.13 (1.97-2.31)
Women	140 (9.5)	160.3	185 637 (3.0)	21.7	2.33 (1.98-2.75)
Other^f^	964 (16.4)	371.7	885 987 (7.2)	65.6	2.00 (1.87-2.13)
Men	863 (19.6)	486.4	727 099 (11.7)	116.5	1.96 (1.83-2.10)
Women	101 (6.9)	114.6	158 888 (2.6)	16.6	2.52 (2.07-3.06)

Abbreviation: HR, hazard ratio.

^a^ Violent assault outcomes: n = 7791; conviction outcomes: n = 5876. People with follow-up ending before age 15 years were excluded from conviction analyses.

^b^ Assault outcomes: n = 13 811 493; conviction outcomes: n = 12 333 904. People with follow-up ending before age 15 years were excluded from conviction analyses.

^c^ The rate was calculated based on the total number of assaults/convictions for the entire follow-up period (where follow-up ended on emigration, December 31, 2013, or death), ie, accounting for repeated assault/offending for each individual. To avoid double counting the same event, hospital/outpatient visit for assault was included only if the elapsed time between 2 consecutive visits was at least 1 year.

^d^ Excluding sexual assault.

^e^ Including sexual crime convictions.

^f^ Other nonviolent crime, eg, fraud, embezzlement, vandalism, and crimes against public activity or order.

When omitting different groups of individuals with comorbid psychiatric disorders from the cohort, one at a time, we found that exclusion of people with ADHD attenuated, but did not completely eliminate the association between TS or CTD and any violent assault experience (aHR, 1.56; 95% CI, 1.32-1.85) (Table 2). Excluding other comorbidities did not affect the association. As shown in Table 3, the associations were attenuated, but the risks remained significantly elevated in the within-sibling analysis (aHR, 1.32; 95% CI, 1.08-1.61).

**Table 2.** Association of Tourette Syndrome or Chronic Tic Disorder With Violent Assault and Criminal Convictions When Excluding Psychiatric Comorbidities

Outcome	Sex- and birth rate–adjusted HR (95% CI)								
	Without exclusion	Exclusion							
		ADHD	Anxiety disorders	Autism spectrum disorders	Conduct disorders	Depressive disorders	OCD	Psychotic disorders	SUDs
Any violent assault	2.21 (2.00-2.43)	1.56 (1.32-1.85)^a^	1.98 (1.73-2.26)	2.29 (2.04-2.56)	2.11 (1.90-2.34)	2.04 (1.81-2.31)	2.21 (1.98-2.47)	2.08 (1.86-2.32)	1.85 (1.63-2.10)
Crime conviction									
Violent	3.13 (2.92-3.36)	2.20 (1.95-2.48)^a^	2.83 (2.57-3.11)	3.04 (2.80-3.31)	2.84 (2.63-3.07)	3.13 (2.88-3.41)	3.14 (2.90-3.40)	2.93 (2.71-3.18)	2.66 (2.42-2.92)
Any nonviolent	1.62 (1.54-1.71)	1.15 (1.06-1.24)^a^	1.40 (1.31-1.50)^a^	1.68 (1.59-1.78)	1.54 (1.46-1.62)	1.54 (1.45-1.63)	1.65 (1.56-1.74)	1.58 (1.50-1.67)	1.34 (1.26-1.42)^a^
Alcohol- or drug-related	2.10 (1.94-2.27)	1.29 (1.12-1.49)^a^	1.81 (1.62-2.02)	2.29 (2.09-2.51)	1.98 (1.82-2.16)	1.91 (1.73-2.12)	2.14 (1.96-2.34)	2.01 (1.84-2.21)	1.39 (1.23-1.58)^a^
Traffic	1.45 (1.34-1.58)	1.08 (0.96-1.23)^a^	1.31 (1.18-1.47)	1.62 (1.48-1.77)	1.41 (1.30-1.54)	1.42 (1.29-1.57)	1.53 (1.40-1.67)	1.50 (1.37-1.64)	1.24 (1.11-1.37)
Property	2.06 (1.92-2.21)	1.35 (1.20-1.53)^a^	1.79 (1.62-1.97)	2.12 (1.95-2.30)	1.95 (1.81-2.11)	2.02 (1.85-2.20)	2.12 (1.96-2.30)	1.97 (1.82-2.14)	1.58 (1.44-1.75)^a^
Other nonviolent^b^	2.00 (1.87-2.13)	1.37 (1.24-1.52)^a^	1.72 (1.58-1.88)	2.03 (1.88-2.18)	1.88 (1.76-2.01)	1.92 (1.77-2.07)	2.06 (1.92-2.21)	1.91 (1.78-2.05)	1.57 (1.44-1.70)^a^

Abbreviations: ADHD, attention-deficit/hyperactivity disorder; HR, hazard ratio; OCD, obsessive-compulsive disorder; SUD, substance use disorder.

^a^ Coefficients indicate attenuation in the estimate compared with the estimate without exclusion for comorbid conditions.

^b^ Other nonviolent crime, eg, fraud, embezzlement, vandalism, and crimes against public activity or order.

**Table 3.** Association of Tourette Syndrome or Chronic Tic Disorder With Violent Assault and Criminal Convictions Within Siblings

Outcome^a^	Sex- and birth year–adjusted HR (95% CI)	
	Between-individual	Within-siblings
Any assault	2.21 (2.00-2.43)	1.32 (1.08-1.61)
Crime conviction		
Violent	3.13 (2.92-3.36)	2.23 (1.86-2.67)
Any nonviolent	1.62 (1.54-1.71)	1.34 (1.20-1.50)
Alcohol- or drug-related	2.10 (1.94-2.27)	1.62 (1.34-1.96)
Traffic	1.45 (1.34-1.58)	1.46 (1.22-1.75)
Property	2.06 (1.92-2.21)	1.48 (1.28-1.71)
Other nonviolent^b^	2.00 (1.87-2.13)	1.56 (1.35-1.80)

Abbreviation: HR, hazard ratio.

^a^ Assault outcomes: families, n = 2 619 160; individuals with Tourette syndrome or chronic tic disorder, n = 5369; unaffected, n = 6 594 830. Conviction outcomes: families, n = 2 138 574; individuals with Tourette syndrome or chronic tic disorder, n = 3732; unaffected, n = 5 435 939.

^b^ Other nonviolent crime, eg, fraud, embezzlement, vandalism, and crimes against public activity or order.

### Risk of Crime Convictions

**Figure 2.**
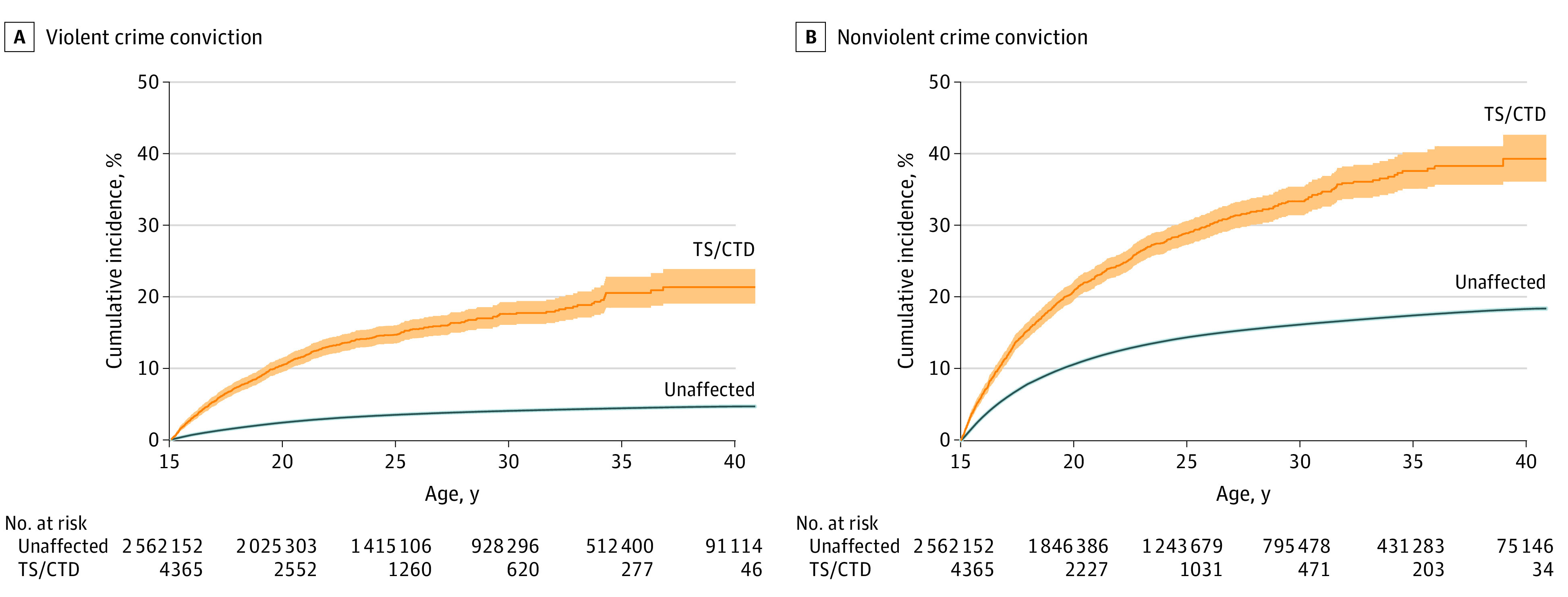
Violent and Nonviolent Crime Conviction in Individuals With Tourette Syndrome (TS) or Chronic Tic Disorder (CTD) Cumulative incidence under the assumption of no competing risks estimated as 1 minus the Kaplan-Meier estimate of survival function for any violent (A) and nonviolent (B) crime convictions in individuals with TS or CTD and unaffected individuals from the general population in the subsample of 2 566 517 individuals followed up from birth. The shaded areas indicate 95% CIs.

We found TS or CTD to be associated with a 3-fold increased risk of violent crime convictions (aHR, 3.13; 95% CI, 2.92-3.36). Nonoverlapping CIs for the estimates suggest that women with TS or CTD had a higher relative risk of convictions for violent offenses than did men (Table 1). Cumulative incidence showed 22% of individuals with TS or CTD having a violent crime conviction by the age of 41 years, compared with 5% in the unaffected general population (Figure 2A).

TS or CTD was associated with a 1.6-fold (aHR, 1.62; 95% CI, 1.54-1.71) increased risk of convictions for any nonviolent offenses (Table 1). In absolute terms, the cumulative incidence of any nonviolent crime conviction was 39% in individuals with TS or CTD, compared with 18% in the general population (Figure 2B).

The association of TS or CTD with violent crime convictions attenuated when individuals with ADHD were excluded, but a 2-fold increased risk persisted even after exclusion (aHR, 2.20; 95% CI, 1.95-2.48) (Table 2). Excluding other comorbidities did not affect the association. A similar pattern emerged for nonviolent convictions, where removing individuals with ADHD partially attenuated the associations, and in the case of convictions for traffic offenses, eliminated the association entirely. Comorbid substance use disorders contributed significantly to the association of TS or CTD with alcohol- and drug-related crime (aHR, 1.39; 95% CI, 1.23-1.58), property crime (aHR, 1.58; 95% CI, 1.44-1.75), and other nonviolent crimes (aHR, 1.57; 95% CI, 1.44-1.70).

Table 3 shows that, when the association of TS or CTD with violent crime convictions was estimated within siblings, the association was reduced, but remained elevated with a more than 2-fold increased risk (aHR, 2.23; 95% CI, 1.86-2.67). A similar pattern was observed for convictions for nonviolent criminal offenses, although the estimates were not as high as those seen in violent offenses (aHR, 1.34; 95% CI, 1.20-1.50).

### Additional Analyses

When the data were restricted to a subsample of individuals with follow-up from birth, the association of TS or CTD with violent assault and conviction outcomes remained similar to the main results (eTable 3 in the Supplement). Among people with TS or CTD, 37.0% (114 of 308; 95% CI, 31.6%-42.4%) of individuals who had experienced any violent assault (sexual and nonsexual) also had a violent crime conviction. The corresponding proportion in the general population was 17.9% (16 067 of 89 920; 95% CI, 17.6%-18.1%). Conversely, 21.3% (114 of 536; 95% CI, 17.8%-24.7%) of those convicted of violent offenses also experienced violence among people with TS or CTD. The corresponding proportion in the general population was 18.6% (16 067 of 86 498; 95% CI, 18.3%-18.8%).

We also estimated the association with an alternative definition for TS or CTD, aiming to capture individuals with a persistent, severe tic disorder. Although some of the point estimates were slightly higher than those observed in the main analysis, the CIs overlapped (eTable 4 in the Supplement).

## Discussion

Results of this cohort study suggest that individuals with TS or CTD had a 2-fold increased risk of experiencing any violent assault (sexual and nonsexual), compared with unaffected individuals from the general population. Exclusion of psychiatric comorbidities, including ADHD, and controlling for unmeasured familial confounders attenuated but did not eliminate the risk. The cumulative incidence was 14% in those followed up from birth, compared with 5% in the general population; patient and control groups started to diverge already before age 15 years, indicating early vulnerability to experiencing violent assault. These results expand those of the previous literature on self- or parent-reported bullying^3,6,39^ by providing new objective data on more serious assault outcomes requiring medical attention.

Results suggest that individuals with TS or CTD had a 3-fold increased risk of violent crime convictions, which decreased to approximately 2-fold after the exclusion of individuals with comorbid ADHD and in the sibling comparison models. These results confirm the results of a previous, much smaller register-based study.^14^ The risk of nonviolent crime convictions (including alcohol- or drug-related crimes, traffic offenses, property crime, and other nonviolent crimes) was comparatively smaller and was substantially attenuated (no longer significant for traffic convictions) when individuals with comorbid ADHD and substance use disorders were excluded from the cohort. In absolute terms, 22% of people with TS or CTD who were followed up from birth had a violent crime conviction by age 41 years (vs 5% of the general population). Similarly, 39% had a conviction for nonviolent crime (vs 18% in the general population). Thus, although most individuals with TS or CTD were not involved in crime, those with comorbid ADHD and substance use disorders may represent a subgroup at greater risk of being involved in crime.

Results of this study further showed that, in line with the previous literature on psychiatric disorders in general,^17^ violent assault and perpetration of crime were not independent phenomena. In our study, 37.0% of the individuals with TS or CTD who had experienced any violent assault also had a violent crime conviction. These findings contribute to our understanding of the origin of disruptive behavior and crime in TS and CTDs.

Further, although men had higher absolute risks of experiencing violent assault and crime convictions, our results showed that the relative risks were highest among women with TS or CTD, probably owing to the lower rates of these outcomes among women from the general population. The reasons for these sex differences are unclear and require further study.

These results have important clinical, social, and medicolegal implications. From a clinical perspective, early detection and management of TS and CTD and associated comorbidities are key. It is paramount to improve access to specialist multidisciplinary teams and to evidence-based treatments, particularly behavior therapy, for TS and CTD.^40^ From a social perspective, more needs to be done to support patient organizations in their efforts to educate the general public about TS and CTD and to reduce the considerable stigma and misunderstanding still associated with these disorders.^4,5^ From a medicolegal perspective, it is important to increase awareness within the legal community on the nature of TS and CTD and associated comorbidities. When appropriate, legal proceedings should involve medical experts who can advise on diminished legal responsibility issues (ie, to help determine if the person’s behavior was deliberately intended to cause harm). A better understanding of the peculiarities of TS and CTD may prevent unwarranted convictions.^10^

### Strengths and Limitations

The main strengths of this study were the large cohort of individuals with TS and CTD who were followed up for a median of 3 decades, thereby providing ample opportunity to capture the outcomes of interest. We systematically accounted for psychiatric comorbidities and adjusted for unmeasured familial confounding. Surveillance bias was likely to be minimal given the seriousness of the outcomes and the fact that the Crime Register is independent from health care.

The study had some limitations. First, the patients were seen in specialist settings, limiting the generalizability of the findings to milder forms of TS and CTD. Second, the violent assault outcomes were serious events requiring medical care, which means that our study could not detect milder or unreported forms of assault, nor did it capture crimes that did not result in convictions. Third, we did not have detailed information on tic symptom severity. We used a proxy for the chronicity of tics, which did not show stronger associations with the outcomes under study. Finally, the sibling comparisons could only adjust for approximately 50% of the shared genetic risks, leaving room for potential residual genetic confounding.

## Conclusions

In this cohort study, results suggest that individuals with TS or CTD diagnosed in specialist settings were more likely to both experience violent assault and be perpetrators of violence. The risk was highest in individuals with comorbid ADHD and substance use disorders. Future studies should focus on understanding the etiology of such serious outcomes in an effort to prevent the experience of assault or criminal convictions in individuals with TS or CTD.

## Notes

Supplement. **eTable 1. ***International Classification of Diseases Codes* for Violent Assault Outcomes and Types of Criminal Offense Outcomes **eTable 2. ***International Classification of Diseases Codes* and Frequencies for Psychiatric Comorbidities **eTable 3. **Association of Tourette Syndrome/Chronic Tic Disorder With Violent Assault and Criminal Convictions Restricted to a Subcohort With Follow-up From Birth **eTable 4. **Association of Persistent Tourette Syndrome/Chronic Tic Disorder With Violent Assault and Criminal Convictions
