# Supplementary material for: Association of Tourette Syndrome and Chronic Tic Disorder With Violent Assault and Criminal Convictions
Source: JAMA Neurol. 2022 Mar 21;79(5):459–67. doi: 10.1001/jamaneurol.2022.0167 (PMC8938899; doi:10.1001/jamaneurol.2022.0167)
Supplement: Supplement. — eTable 1. International Classification of Diseases Codes for Violent Assault Outcomes and Types of Criminal Offense Outcomes eTable 2. International Classification of Diseases Codes and Frequencies for Psychiatric Comorbidities eTable 3. Association of Tourette Syndrome/Chronic Tic Disorder With Violent Assault and Criminal Convictions Restricted to a Subcohort With Follow-up From Birth eTable 4. Association of Persistent Tourette Syndrome/Chronic Tic Disorder With Violent Assault and Criminal Convictions [file jamaneurol-e220167-s001.pdf]

Supplementary Online Content

Mataix-Cols D, Virtanen S, Sidorchuk A, et al. Association of Tourette syndrome and chronic tic disorder with violent assault and criminal convictions. *JAMA Neurol*. Published online March 21, 2022. doi:10.1001/jamaneurol.2022.0167

eTable 1. *International Classification of Diseases Codes* for Violent Assault Outcomes and Types of Criminal Offense Outcomes

eTable 2. *International Classification of Diseases Codes* and Frequencies for Psychiatric Comorbidities

eTable 3. Association of Tourette Syndrome/Chronic Tic Disorder With Violent Assault and Criminal Convictions Restricted to a Subcohort With Follow-up From Birth

eTable 4. Association of Persistent Tourette Syndrome/Chronic Tic Disorder With Violent Assault and Criminal Convictions

This supplementary material has been provided by the authors to give readers additional information about their work.

© 2022 Mataix-Cols D et al. *JAMA Neurology.*

eTable 1. International Classification of Diseases Codes for Violent Assault Outcomes and Types of Criminal Offense Outcomes

	**ICD code**			
**Outcome**	**ICD-8**	**ICD-9**	**ICD-10**	**Offense types**
Any violent assault	E960–E969	E960–E969	X85–Y05	
Sexual assault		E960.1	Y05, T74.2	
Violent crime				Murder, manslaughter, assault, kidnapping, illegal restraint, illegal coercion or threats, robbery, threats or violence against an officer, arson, gross violation of a person's integrity, harassment, rape, sexual coercion, child molestation, sexual intercourse with a child, child pornography offenses, pimping, sexual harassment
Alcohol/drug-related crime				Driving under the influence of alcohol or drugs; Violations of the Narcotic Drugs Act, such as possession, manufacturing, trafficking or sales of narcotics
Traffic-related crime				Reckless driving, hit and run offenses, causing death or injury by driving, moving violations
Property crimes				Theft, burglary, appropriation of a vehicle, unlawful dispossession, unlawful repossession, unlawful diversion of power
Other non-violent crimes				Any non-violent criminal offense not included in other categories

© 2022 Mataix-Cols D et al. *JAMA Neurology.*

eTable 2. International Classification of Diseases Codes and Frequencies for Psychiatric Comorbidities

	ICD-codes			Frequencies (%)	
Comorbidities	ICD-8	ICD-9	ICD-10	People with TS/CTD (n=7,791)	Unaffected general population (n=13,811,493)
ADHD	-	314	F90	3,971 (51.0)	152,180 (1.1)
Anxiety disorders	300.00, 300.20, 307, 308.4	300A, 300C, 308, 309	F40, F41, F43	1,992 (25.6)	580,538 (4.2)
Autism spectrum disorders	–	299	F84.0, F84.1, F84.5, F84.8, F84.9	2,007 (25.8)	52,030 (0.4)
Conduct disorders	-	-	F91	543 (7.0)	10,920 (0.1)
Depressive disorders	296.0, 296.2, 298.09	296B, 296X, 298A, 311, 300E	F32-F34 (excl. F34.0), F38-F39	1,554 (20.0)	566,971 (4.1)
OCD	300.3	300D	F42	1,348 (17.3)	36,308 (0.3)
Psychotic disorders	295, 296.00, 296.10, 296.30, 296.88, 296.99, 297, 298	295, 296A, 296C, 296E, 296W, 296X, 297, 298	F20-F25, F28, F29, F30-F31, F34.0	718 (9.2)	263,769 (1.9)
Substance use disorders	303, 304, 291, 294.30	303, 304, 305A, 305X, 291, 292	F10-F16, F18-F19	892 (11.5)	491,145 (3.6)

Note: ADHD was also defined as ever having dispensed stimulant medication with Anatomical Therapeutic Chemical (ATC) classification codes N06BA01, N06BA02, N06BA04, N06BA09, and N06BA12. Psychotic disorders include bipolar disorders and schizophrenia spectrum disorders.

Abbreviations: ADHD attention-deficit/hyperactivity disorder, ICD International Classification of Diseases, OCD obsessive-compulsive disorder.

© 2022 Mataix-Cols D et al. *JAMA Neurology.*

eTable 3. Association of Tourette Syndrome/Chronic Tic Disorder With Violent Assault and Criminal Convictions Restricted to a Subcohort With Follow-up From Birth

	**People with Tourette syndrome or chronic tic disorder [a]**	**Unaffected general population [b]**	
**Outcome**	**n (%)**	**n (%)**	**HR (95% CI) adjusted for sex and birth year**
**Violent assault**			
**Any assault**	308 (5.0)	89,920 (2.4)	2.02 (1.81-2.26)
Men	244 (5.0)	60,067 (3.1)	1.86 (1.64-2.11)
Women	64 (4.9)	29,853 (1.6)	3.18 (2.49-4.06)
Sexual assault[c]	37 (0.6)	12,501 (0.3)	2.91 (2.11-4.02)
Nonsexual assault[d]	276 (4.5)	79,113 (2.1)	1.96 (1.75-2.21)
Men	237 (4.9)	58,008 (3.0)	1.86 (1.63-2.11)
Women	39 (3.0)	21,105 (1.1)	2.84 (2.07-3.89)
**Conviction for criminal offense**			
**Violent crime conviction [e]**	537 (12.3)	86,539 (3.4)	3.49 (3.21-3.80)
Men	487 (14.3)	73,548 (5.6)	3.41 (3.12-3.72)
Women	50 (5.3)	12,991 (1.0)	5.63 (4.26-7.43)
**Any non-violent crime conviction**	1,036 (23.7)	355,947 (13.9)	1.85 (1.74-1.96)
Men	895 (26.2)	262,523 (20.0)	1.82 (1.71-1.95)
Women	141 (14.8)	93,424 (7.5)	2.36 (2.00-2.78)
Alcohol/drug-related crime conviction	395 (9.1)	98,712 (3.9)	2.21 (2.01-2.44)
Men	358 (10.5)	82,673 (6.3)	2.14 (1.93-2.37)
Women	37 (3.9)	16,039 (1.3)	3.54 (2.57-4.89)
Traffic crime conviction	327 (7.5)	122,800 (4.8)	1.59 (1.42-1.77)
Men	309 (9.1)	110,201 (8.4)	1.55 (1.38-1.73)
Women	18 (1.9)	12,599 (1.0)	2.52 (1.59-4.00)
Property crime conviction	507 (11.6)	145,406 (5.7)	2.32 (2.12-2.53)
Men	418 (12.3)	91,074 (6.9)	2.40 (2.18-2.64)
Women	89 (9.4)	54,332 (4.4)	2.43 (1.97-2.99)
Other non-violent crime conviction	643 (14.7)	178,265 (7.0)	2.22 (2.06-2.40)
Men	583 (17.1)	145,183 (11.1)	2.19 (2.02-2.37)
Women	60 (6.3)	33,082 (2.7)	2.91 (2.26-3.75)

Notes: [a]Assault outcomes n=6,165; Conviction outcomes n=4,365; People with follow-up ending before age 15 were excluded from conviction analyses.

[b]Assault outcomes n=3,828,057; Conviction outcomes n=2,562,152; People with follow-up ending before age 15 were excluded from conviction analyses.

[c] Models were not estimated for men and women separately due to insufficient number of cases.

[d] Excluding sexual assault

[e] Including sexual crime convictions

© 2022 Mataix-Cols D et al. *JAMA Neurology.*

eTable 4. Association of Persistent Tourette Syndrome/Chronic Tic Disorder With Violent Assault and Criminal Convictions

	**People with Tourette syndrome or chronic tic disorder [a]**	**Unaffected general population [b]**	
**Outcome**	**n (%)**	**n (%)**	**HR (95% CI) adjusted for sex and birth year**
**Violent assault**			
Any assault	197 (9.0)	254,895 (1.8)	2.68 (2.33-3.08)
Men	143 (9.2)	146,000 (2.1)	2.23 (1.89-2.62)
Women	54 (8.4)	108,805 (1.6)	3.77 (2.89-4.93)
Sexual assault[c]	37 (1.7)	45,594 (0.3)	4.65 (3.37-6.43)
Nonsexual assault[d]	171 (7.8)	214,187 (1.6)	2.53 (2.18-2.94)
Men	138 (8.9)	131,179 (1.9)	2.22 (1.88-2.62)
Women	33 (5.1)	83,008 (1.2)	2.99 (2.12-4.21)
**Conviction for criminal offense**			
**Violent crime conviction [e]**	393 (18.1)	322,720 (2.6)	3.61 (3.27-3.98)
Men	344 (22.4)	284,990 (5.0)	3.41 (3.07-3.80)
Women	49 (7.7)	37,730 (0.6)	7.21 (5.45-9.54)
**Any non-violent crime conviction**	751 (34.5)	1,757,905 (14.3)	1.73 (1.61-1.86)
Men	614 (39.9)	1,341,662 (21.6)	1.66 (1.54-1.80)
Women	137 (21.4)	416,243 (6.8)	2.42 (2.05-2.86)
Alcohol/drug-related crime conviction	323 (14.8)	436,651 (3.5)	2.45 (2.20-2.74)
Men	280 (18.2)	381,178 (6.1)	2.31 (2.06-2.60)
Women	43 (6.7)	55,473 (0.9)	4.81 (3.57-6.48)
Traffic crime conviction	283 (13.0)	769,370 (6.2)	1.48 (1.32-1.66)
Men	261 (17.0)	654,386 (10.6)	1.45 (1.28-1.63)
Women	22 (3.4)	114,984 (1.9)	1.64 (1.08-2.50)
Property crime conviction	376 (17.3)	567,003 (4.6)	2.30 (2.08-2.54)
Men	289 (18.8)	381,366 (6.2)	2.23 (1.99-2.50)
Women	87 (13.6)	185,637 (3.0)	2.93 (2.37-3.62)
Other non-violent crime conviction	487 (22.4)	885,987 (7.2)	2.10 (1.92-2.29)
Men	420 (27.3)	727,099 (11.7)	2.01 (1.82-2.21)
Women	67 (10.5)	158,888 (2.6)	3.04 (2.39-3.87)

Notes: [a]Assault outcomes n=2,197; Conviction outcomes n=2,177; People with follow-up ending before age 15 were excluded from conviction analyses. Restricted to individuals with persistent tics, defined as at least two visits to psychiatric specialist services for Tourette syndrome or chronic tic disorder after age 18.

[b]Assault outcomes n=13,811,493; Conviction outcomes n=12,333,904; People with follow-up before age 15 only were excluded from conviction analyses.

[c] Models were not estimated for men and women separately due to insufficient number of cases.

[d] Excluding sexual assault

[e] Including sexual crime convictions

© 2022 Mataix-Cols D et al. *JAMA Neurology.*
